# Supplementary material for: LUX ARRHYTHMO mediates crosstalk between the circadian clock and defense in Arabidopsis
Source: Nat Commun. 2019 Jun 11;10:2543. doi: 10.1038/s41467-019-10485-6 (PMC6560066; doi:10.1038/s41467-019-10485-6)
Supplement: Supplementary file 1 — Supplementary Information [file 41467_2019_10485_MOESM1_ESM.pdf]

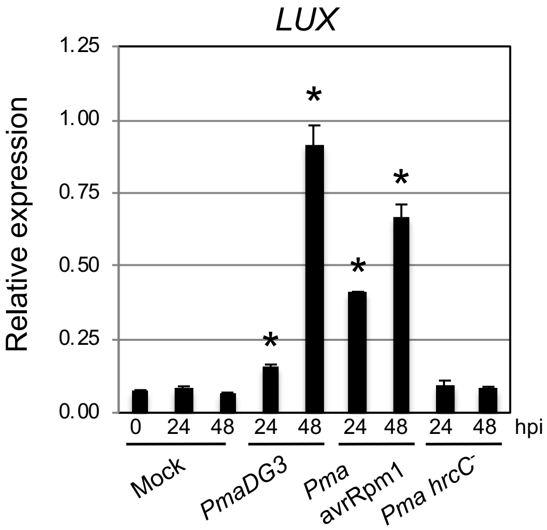

Supplementary Figure 1. Expression of *LUX* is induced by *P. syringae* infection. Total RNA was extracted from 25-d old plants at the indicated hour post infiltration with *P. syringae* strains. Statistical analysis was performed by One-way ANOVA with post-hoc Tukey HSD test. Asterisks indicate significant difference in *LUX* expression between the infected and mock-treated samples at the same time point (n=2; $P<0.05$). These experiments were repeated two times and similar results were obtained.

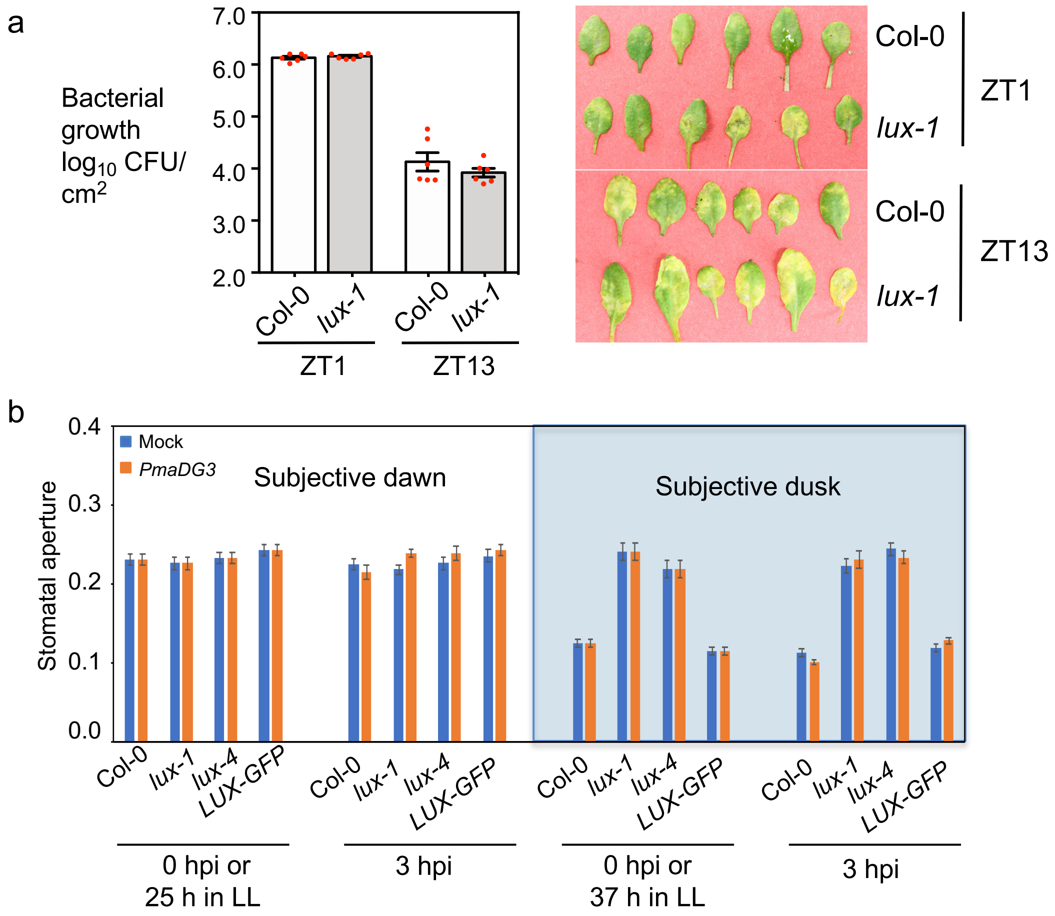

Supplementary Figure 2. *lux*-conferred *P. syringae* susceptibility is influenced by light and stomata. a Bacterial growth (left) and pictures (right) of infected leaves. Plants grown in LD with a light intensity of 180 μmol m^{-2} s^{-1} and spray-infected with *PmaDG3* (OD = 0.01) 1 or 13 h after transfer to LL (n =6). **b** Stomatal aperture measurement (n=80). LD (180 μmol m^{-2} s^{-1} light intensity) entrained 25-day old plants were transferred to LL (10 μmol m^{-2} s^{-1} light intensity) for one day. The fifth to seventh leaves of each genotype taken at LL25 or LL37 were incubated with *PmaDG3* (OD_{600}=0.1) or sterile water for 0, 1, or 3 hpi followed by immediate processing for stomata imaging. The stomatal aperture was determined by the ratio between the width and the length of a stoma. Data are presented as mean ± SEM. Statistical analysis was performed with One-way ANOVA post-hoc Tukey HSD test. These experiments were repeated two times and similar results were obtained.

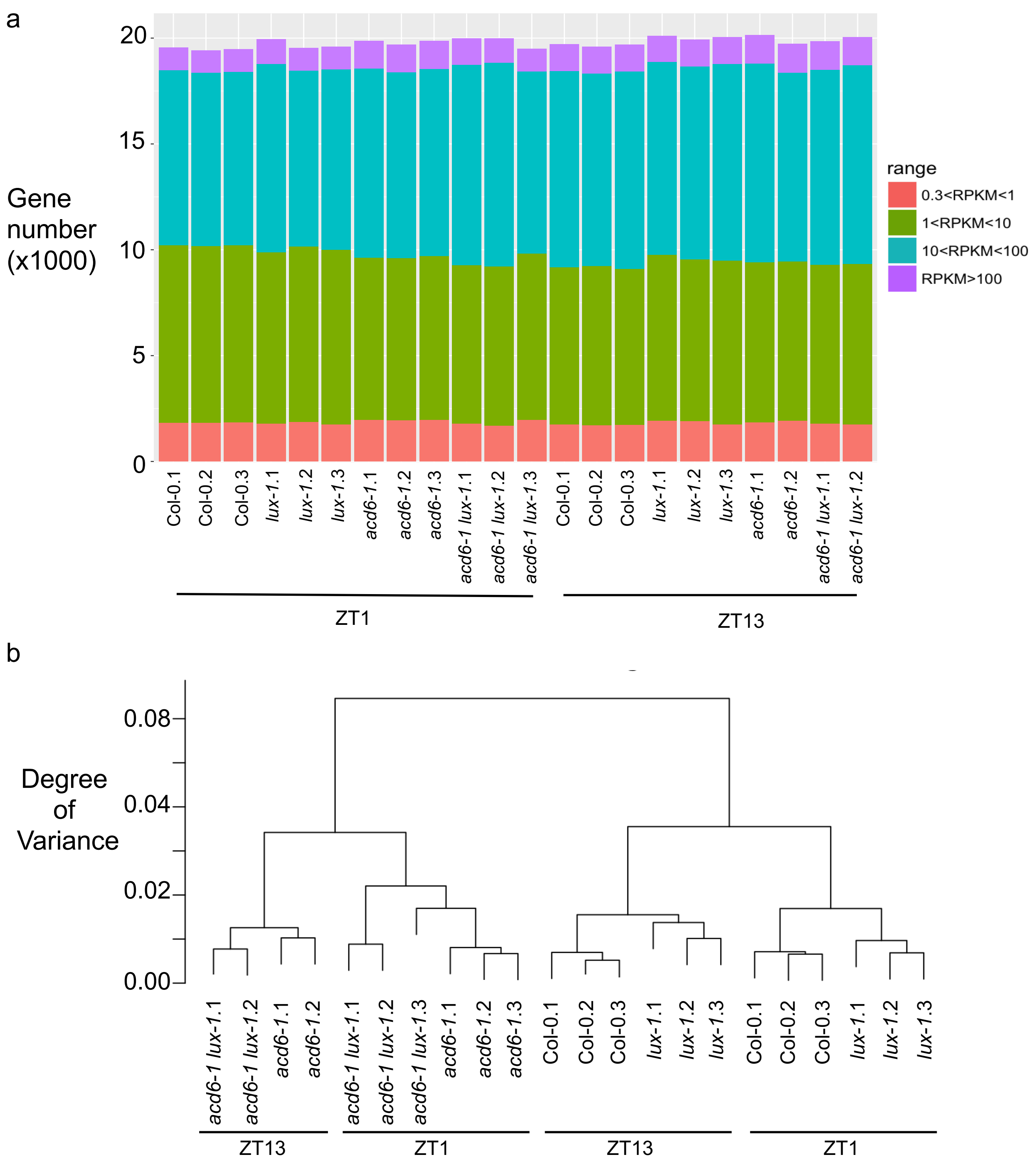

Supplementary Figure 3. Global gene expression analysis of RNA-seq data. a Number of genes expressed in each sample. The relative expression value RPKM of higher than 0.3 was used as a cutoff to define expressed genes. **b** Cluster dendrogram analysis shows global gene expression profiles in biological replicates.

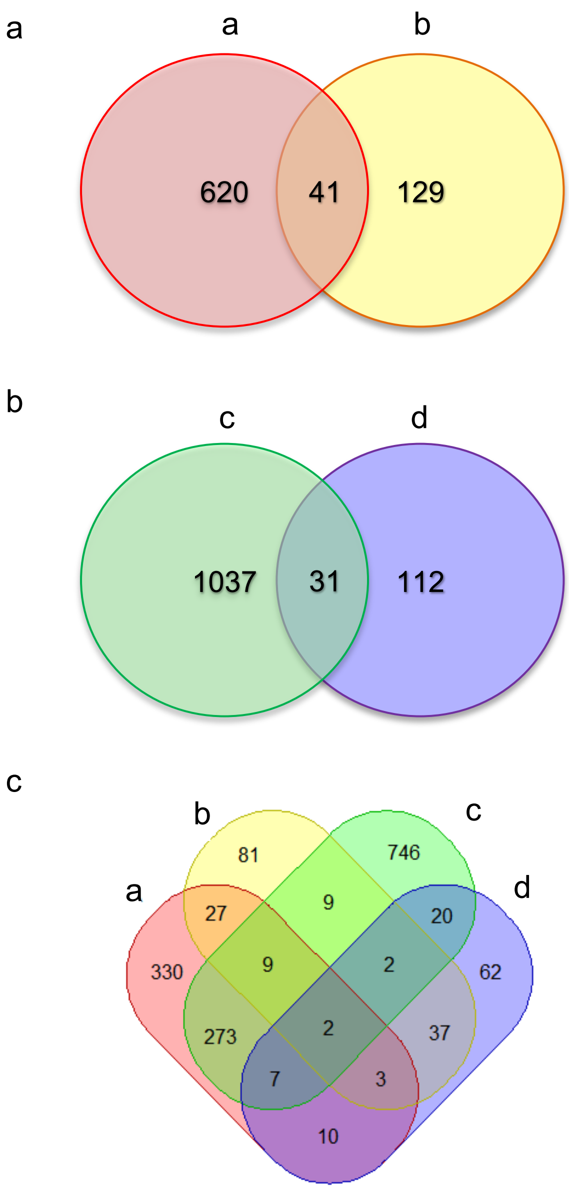

Supplementary Figure 4. Venn diagrams show the number of genes affected by *lux-1*. The comparison groups are: (a) Col-0 vs. *lux-1* at ZT1; (b) Col-0 vs. *lux-1* at ZT13; (c) *acd6-1* vs. *acd6-1lux-1* at ZT1; and (d) *acd6-1* vs. *acd6-1lux-1* at ZT13. **a** Genes affected by *lux-1* under non-defense conditions (the Col-0 background; a & b). **b** Genes affected by *lux-1* under defense conditions (the *acd6-1* background; c & d). **c** Genes affected by *lux-1* in all four comparison groups.

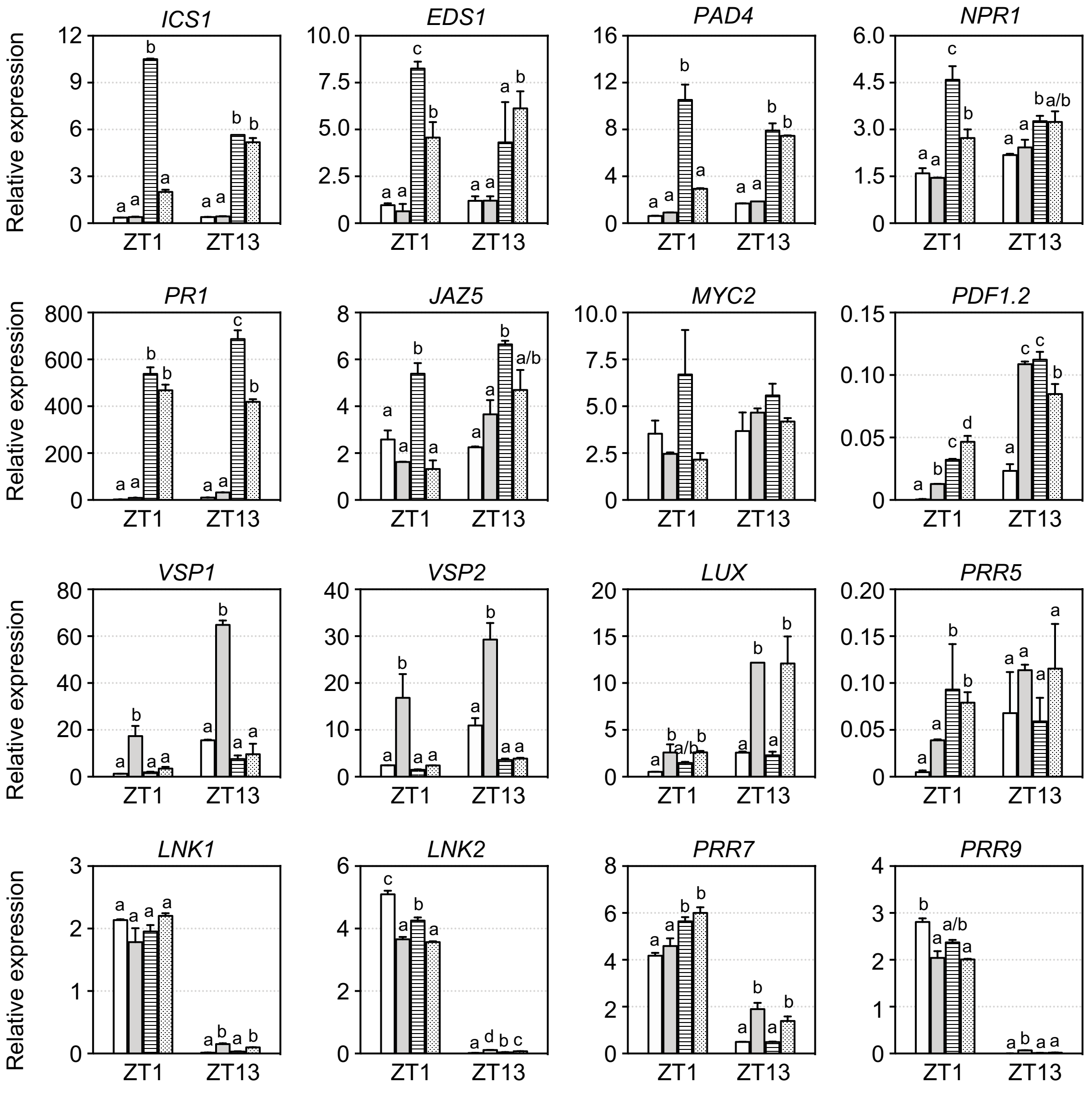

Supplementary Figure 5. Quantification of gene expression by qRT-PCR in Col-0, *acd6-1*, *lux-1*, and *acd6-1lux-1*. 25-d old plants of each genotype grown in LD were collected at ZT1 or ZT13 for RNA extraction followed by qRT-PCR analysis. Data are presented as mean (n=2) ± SD. Statistical analysis was done by One-way ANOVA with post-hoc Tukey HSD test. Different letters indicate significant difference among the samples at the same time point ($P<0.05$). These experiments were repeated two times and similar results were obtained.

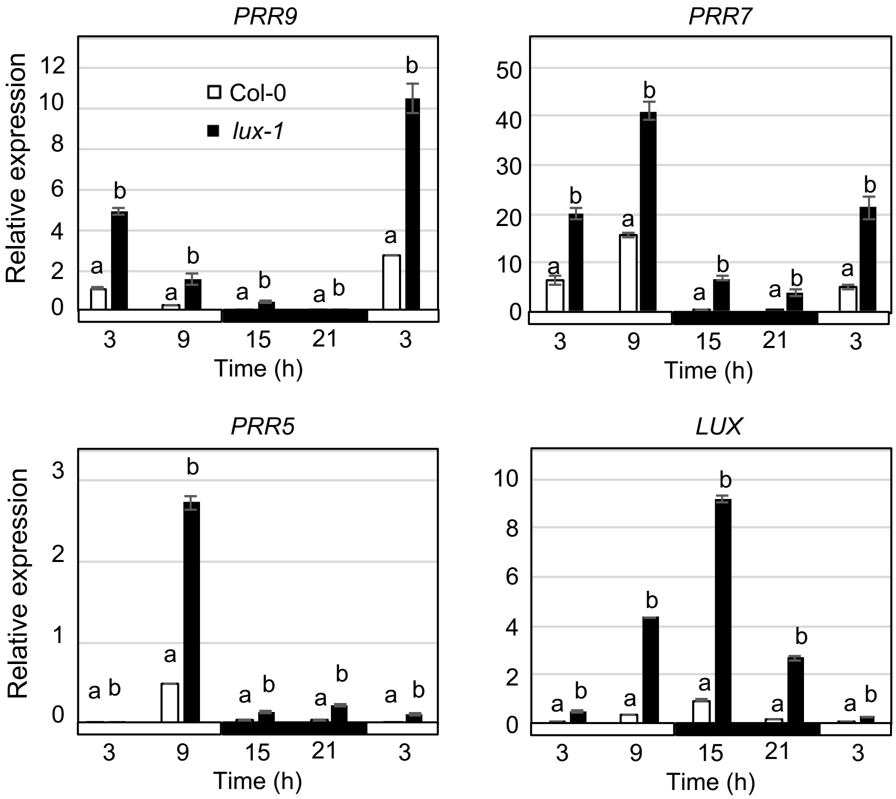

Supplementary Figure 6. Rhythmic clock gene expression in Col-0 and *lux-1* in LD. 25-d old plants of each genotype grown in LD were collected at the indicated time for RNA extraction followed by qRT-PCR analysis. Data are presented as mean (n=2) ± SD. Statistical analysis was done by One-way ANOVA with post-hoc Tukey HSD test. Different letters indicate significant difference among the samples at the same time point ($P<0.05$). Light and dark bars beneath the graphs indicate day and night, respectively, in the light-dark (LD) cycle. These experiments were repeated two times and similar results were obtained.

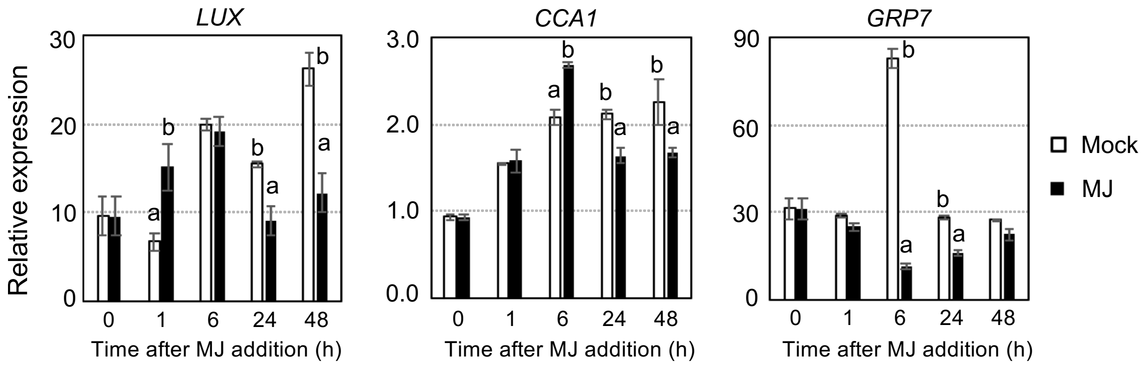

Supplementary Figure 7. MJ treatment suppresses clock gene expression in seedlings. 6-d-old seedlings grown in LD were transferred to sterile water in a 24-well plate and kept in LL for 1 d. The seedlings were treated at 25 h after the onset of LL with 100 µM MJ and were harvested at the indicated times post treatment for RNA extraction followed by qRT-PCR analysis. Data are presented as mean ± SD (n=2). Statistical analysis was done by One-way ANOVA with post-hoc Tukey HSD test. Different letters indicate significant difference among the samples at the same time point ($P<0.05$). These experiments were repeated two times and similar results were obtained.

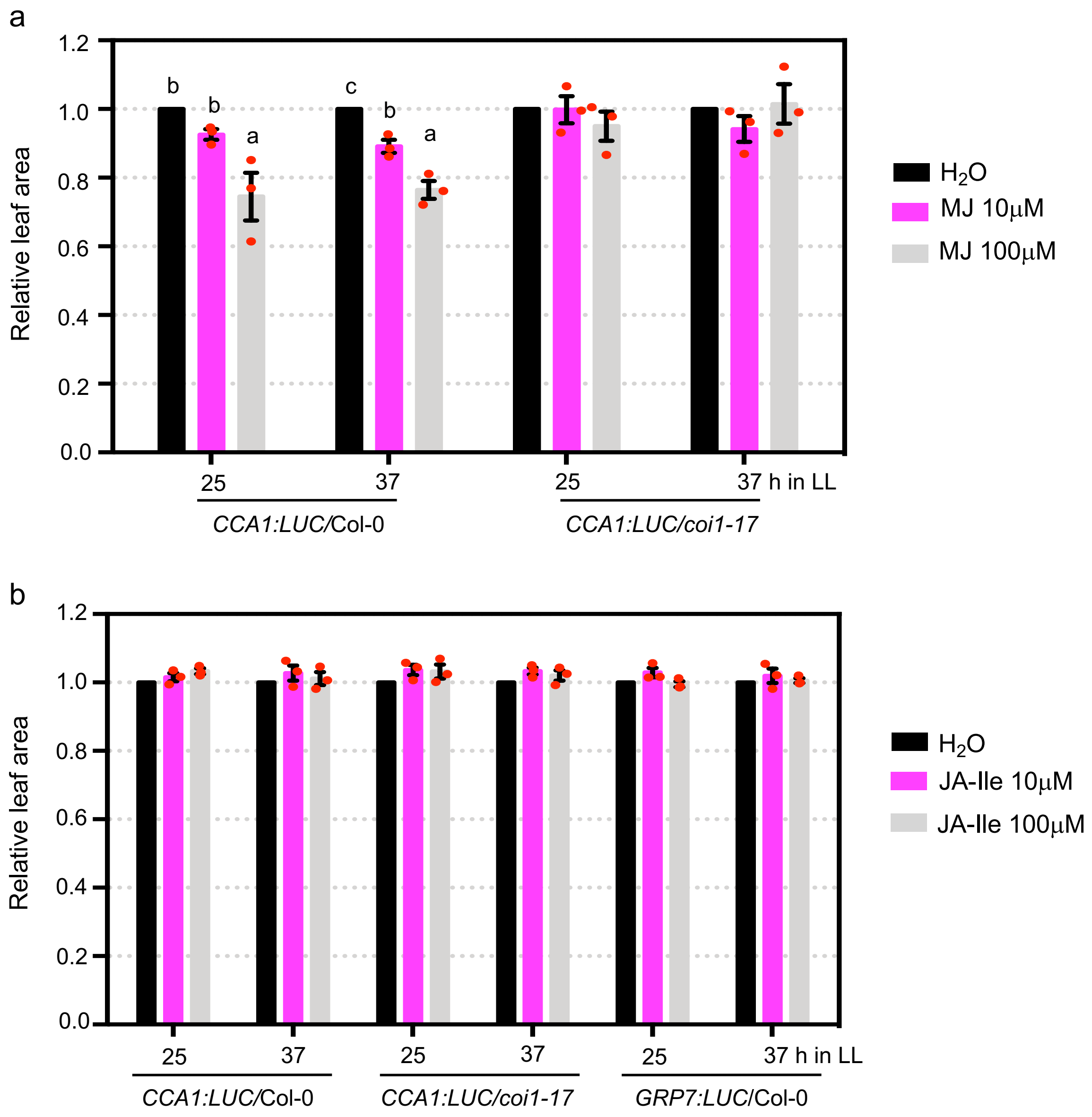

Supplementary Figure 8. Seedling growth inhibition by MJ treatment. a Relative seedling leaf area with MJ treatment. **b** Relative seedling leaf area with JA-Ile treatment. Seedlings used in the luciferase assay were photographed at the end of luminescence recording. The area of each seedling was measured with ImageJ. The average leaf area of mock-treated samples of each genotype was set to 1 and used to calculate the relative leaf area of seedlings of the same genotype that were chemically treated at the same time. Data represent mean (± SEM) of three independent experiments (n=8 or 12 for each experiment). Statistical analysis was performed by One-way ANOVA post-hoc Tukey HSD test. Different letters indicate significant difference among the samples treated at the same time point, 25 h or 37 h after LL ($P<0.05$).

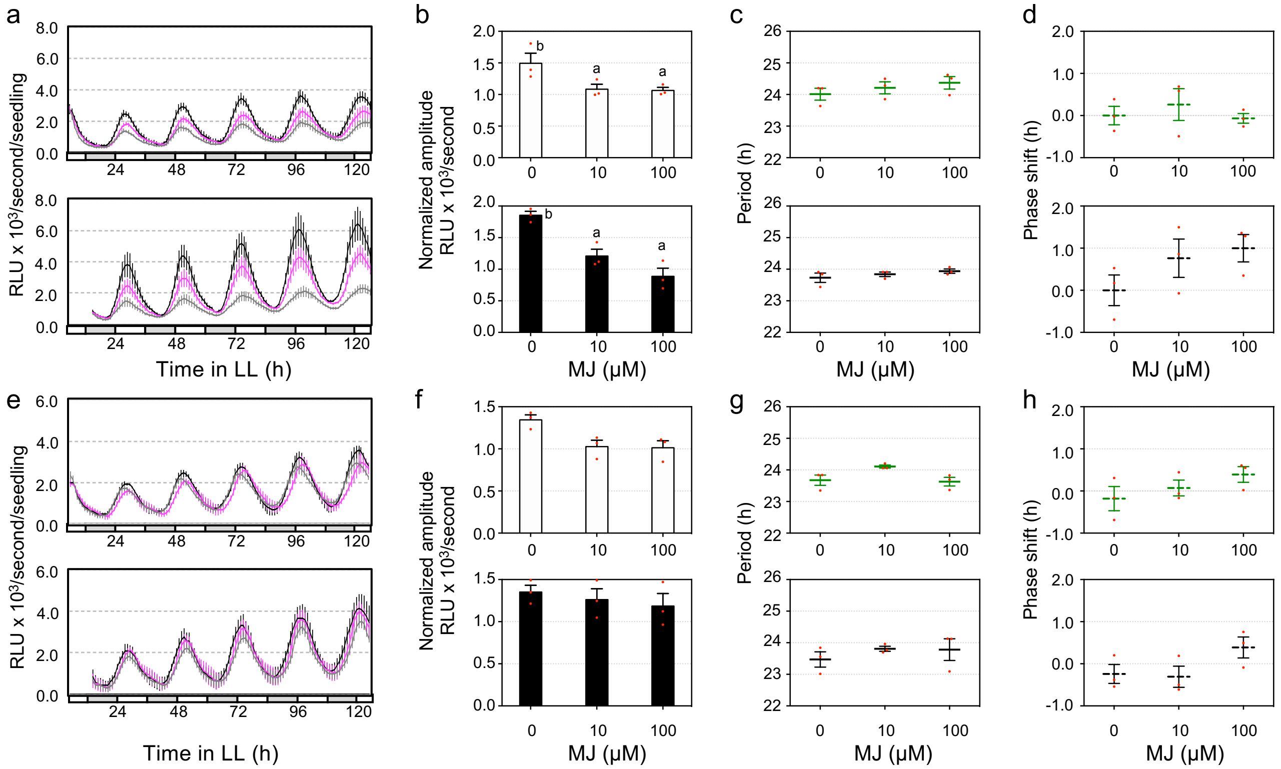

Supplementary Figure 9. MJ treatment affects clock activity. Five-day old seedlings entrained in LD were transferred to LL for 1 d and were treated with MJ. Luminescence was recorded at 1-h intervals for five days and analyzed for amplitude, period, and phase with the R package MetaCycle. **a-d** Expression of *CCA1:LUC* in Col-0 treated with MJ 25 h (top) or 37 h (bottom) after onset of LL. **e-h** Expression of *CCA1:LUC* in *coi1-17* treated with MJ 25 (top) or 37 (bottom) h after onset of LL. **a** and **e** Luminescence traces. RLU: Relative luminescence units. The color indicates MJ concentration, black for 0 , magenta for 10 μM, and gray for 100 μM. **b** and **f** Normalized amplitude. The amplitude of the reporter was normalized to the relative leaf area shown in Supplementary Figure 8. **c** and **g** Period. **d** and **h** Phase shift. Data represent mean (± SEM) of three independent experiments (n=8 or 12 for each experiment). Statistical analysis was performed by One-way ANOVA post-hoc Tukey HSD test. Different letters indicate significant difference among the samples ($P<0.05$).

Supplementary Table 1. ChIP analysis of selected LBS motifs of LUX-affected genes. The positions of LBS motifs within the 1500 bp-promoter region, relative to the transcription start site, of selected LUX-affected genes were predicted via bioinformatics analysis and were indicated in parentheses []. ChIP experiments were conducted with the LBS motifs highlighted in color. Magenta indicates a detection of LUX binding to the motif and blue for no LUX binding in our experiments. Black indicates that motifs need to be tested.

AGI	Name	sense (GATTCG)	anti-sense (CGAATC)	sense (GATACG)	anti-sense (CGTATC)
AT1G17380	JAZ5	[]	[]	[927]	[856]
AT1G30135	JAZ8	[]	[957, 625]	[507]	[]
AT1G32640	MYC2	[]	[1475]	[]	[]
AT1G74710	ICS1	[]	[393]	[]	[607]
AT2G14610	PR1	[977]	[]	[455, 223]	[]
AT2G34600	JAZ7	[607]	[1550]	[]	[]
AT2G38470	WRKY33	[]	[]	[713]	[]
AT3G48090	EDS1	[]	[936]	[]	[]
AT3G52430	PAD4	[110]	[]	[]	[1068]
AT5G20480	EFR	[612]	[]	[819]	[]
AT1G22770	GI	[]	[1074]	[]	[]
AT2G46790	PRR9	[]	[172]	[]	[]
AT3G46640	LUX	[]	[367]	[]	[]
AT3G54500	LNK2	[]	[]	[74]	[]
AT5G02810	PRR7	[1020]	[]	[1127]	[595]
AT5G15840	CO	[723, 47]	[360]	[311]	[1234]
AT5G60100	PRR3	[]	[646]	[]	[950]
AT5G62430	CDF1	[]	[]	[]	[30]
AT5G64170	LNK1	[492]	[701, 500]	[]	[]

Supplementary Table 2. Primers used in this report. Position of LBS motif indicates the location of each LBS site relative to the transcription start site of the gene.

**Genotyping primers**			
	**Primer names**	**Primer sequence (5 > 3')**	
acd6-1	acd6 d*TMboII	GCCATTTCACATGGGCAATTGCAGTG ATCACGCCAAAGA	
	acd6_dCAPSfor	CTTCATTTTCTGCTTTTGACATCTT G	
lux-1	lux-1 NalIII dCaps-F	TTCCGGCGACGGAGATAGGGTTTCTG CAT	
	lux-1 dCaps-R	ACCACGTAGCGACGAGAGCGT	
**qRT-PCR primers**			
LUX	LUX-qPCR-f	AACACCTGTTCCTCCACAGAGC	
	LUX-qPCR-r	TCCAACATTACCGCTGCTACCG	
ICS1	SID2-qPCR-f	GCTTGGCTAGCACAGTTACAGC	
	SID2-qPCR-r	CACTGCAGACACCTAATTGAGTCC	
EDS1	EDS1-qPCR-f	GCTCAATGACCTTGGAGTGAGC	
	EDS1-qPCR-r	TCTTCCTCTAATGCAGCTTGAACG	
PAD4	PAD4-qPCR-f	AGATACGCGAGCACAACGCAAG	
	PAD4-qPCR-r	TTCTCGCCTCATCCAACCACTC	
NPR1	NPR1-qPCR-f	AACGATTCTTCCCGCGCTGTTC	
	NPR1-qPCR-r	TTCTCCGCAAGCCAGTTGAGTC	
PRR5	PRR5-qPCR-f	AGCTTTCACACGGTACGTTCAC	
	PRR5-qPCR-r	TTGGAGGCGGTTCAGATGTATTG	
PRR7	PRR7-qPCR-f	AAGCGGAAGTGGAAGTGGTAGC	
	PRR7-qPCR-r	TCCGGCTTTGGTATCGTACCTTC	
PR1	PR1-qPCR-f	ACACGTGCAATGGAGTTTGTGG	
	PR1-qPCR-r	TTGGCACATCCGAGTCTCACTG	
JAZ5	JAZ5-qPCR-f	TTCCAAAGGCGAACCCTCTACC	
	JAZ5-qPCR-r	TCCTGGCTGTGATTCACTGAGG	

PDF1.2	PDF1.2-qPCR-f	CTTGTTCTCTTTGCTGCTTTCGAC	
	PDF1.2-qPCR-r	TTGGCTCCTTCAAGGTTAATGCAC	
VSP1	VSP1-qPCR-f	TCGAGAATCTCAAGGCTGTTGGTG	
	VSP1-qPCR-r	TCAACTTCGATCCGTTTGGCTTG	
VSP2	VSP2-qPCR-f	GGACTTGCCCTAAAGAACGACACC	
	VSP2-qPCR-r	GTCGGTCTTCTCTGTTCCGTATCC	
LNK1	LNK1-qPCR-f	GGATGTGGACAACATGCTTAGGAG	
	LNK1-qPCR-r	TTTGGCTGGGCAGAAGAGAACC	
LNK2	LNK2-qPCR-f	GCAGAATTCGCAGTTCTTTATCGG	
	LNK2-qPCR-r	CCGTGTTCCCATATCCAACTTTGC	
MYC2	MYC2-qPCR-f	AACCACGTCGAAGCAGAGAGAC	
	MYC2-qPCR-r	TTGGTACAACCGCTCGTAACGC	
**Primers used for ChIP assays**			**Position of LBS motif**
PRR3	PRR3-chip-f1	GTGGTAGAGAATGCGCTCGT	950
	PRR3-chip-r1	GCCTTCTTCTGTGCCGCTAT	
PRR7	PRR7-chip-f1	GTTCCTGAGGCAGAGTTGGT	1020, 1127
	PRR7-chip-r1	TGTTCCTGAGCTCGACCGTA	
PRR7	PRR7-chip-f2	GTAGATAGCCACGACGACGG	595
	PRR7-chip-r2	TGCGTTTCTAAGTCTACCCCT	
PRR9	PRR9-chip-f	CCTGCGAAGCAGAGGACCACC	172
	PRR9-chip-r	AGCGGGCCTTCACTGAGCTG	
GI	GI-chip-f1	AGCCCATCACATCACATGGT	1074
	GI-chip-r1	GGACGTTGATCACTCGCCAA	
CDF1	CDF1-chip-f1	AGAGGCCACATAAACTTGTGGT	30
	CDF1-chip-r1	TCTGACTCTCTGTGTCTCTTTTCC	
CDF1-5'	CDF1-5'-f	TGA CCA AGA TGC AGG TGA GT	
	CDF1-5'-r	ACC AAA AAG GTC TCT GCC ACA	

CDF1-3'	CDF1-3'-f	TCA TTG TCT TGC ATC AGA ACC A	
	CDF1-3'-r	CCA TGC TGT TGC ATC TTG GAC	
LUX	LUX-chip-f	TCCAACGGTGGAAAGATCACATTGC	367
	LUX-chip-r	GTTCGGCCAGCTGGAGAGTG	
CO	CO-chip-f1	CTCATGTGGACTCCAAAATGCC	723
	CO-chip-r1	ATGAGAATCATATCGGAAAAGTGAC	
CO	CO-chip-f2	ACAGTAATCACAAGGTTCAGGA	311, 360
	CO-chip-r2	CCATGGTGTTGCAGGCAAAT	
LNK1	LNK1-chip-f1	CCTTGATTGTTCGGGCTTGC	492, 500
	LNK1-chip-r1	GGGAGCGATGAGAGATGGAA	
LNK1-3'	LNK1-3'-f	ATA CAA TCT CTT CTA TCT GCT GAG T	
	LNK1-3'-r	GCC GAA CGA AAT AAG CTC ACC	
LNK1-5'	LNK1-5'-f	TCT CCA CAA ACT CAG GAG TAC AA	
	LNK1-5'-r	GTT GGG AAA AAG CGA CGT GA	
LNK2	LNK2-chip-f1	ACCACAGCCAGGGAAAGTCA	74
	LNK2-chip-f2	CAAACGAGTGGCTGAGAGAGA	
LNK2-3'	LNK2-3'-f	AGC TGA GCA AAA GAT AAC TGG GA	
	LNK2-3'-r	AAA TCT GGC TGA GGA AGC CC	
LNK2-5'	LNK2-5'-f	AAA AAT TGT TGA ATT GGT GGG AAA A	
	LNK2-5'-r	CCT TTC GTT CAT CAA AGT CCA CA	
ICS1	SID2-chip-f1	CGAGAACTTGTAATGCGTTTGC	607
	SID2-chip-r1	GATACGGAAGCGGTTTGCAC	
PR1	PR1-chip-f1	TTCAGCCATAGGCAAGAGTGA	455
	PR1-chip-r1	ATCGTATCGGACAGTTTGGC	
PR1	PR1-chip-f2	CAAAACAACTGAATGACATGAAACA	223
	PR1-chip-r2	TGCAATTGTCCAAATGAATAGAAGT	
EDS1	EDS1-chip-f1	ACCGTTCATTACACGAAGACA	936

	EDS1-chip-r1	GGGGAAATTTCTACGAAAAGCCA	
EDS1-5'	EDS1-5'-f	GAG CTG GCC ATT TTC TGT ATC C	
	EDS1-5'-r	TCA GCT CCT TCT TCA AGA CAT C	
EDS1-3'	EDS1-3'-f	TGA GTC TCC AAT AGC CAA AGA GT	
	EDS1-3'-r	ACC CCA TCA TGA GAC CAT TTC AA	
PAD4	PAD4-chip-f1	CGAGCTGTATTATTAAGGTGGAAGA	1068
	PAD4-chip-r1	TTTGCTCTAACATGCTTCTTTTCA	
PAD4	PAD4-chip-f2	AAACTCTTACCAAAGTTTCTGCAT	110
	PAD4-chip-r2	AGAAGAAAATGCGAATCAAAGCA	
EFR	EFR-chip-f1	TTGCAGAAGCAAATCTATCTGAACT	819
	EFR-chip-r1	CGTCGACGAAGGAGAAAAACAAT	
EFR	EFR-chip-f2	TCACAGTGACAACACTTCCCAA	612
	EFR-chip-r2	GGAAGCCCATCGATTCTTGC	
GRX480	GRX480-chip-f1	AGGAACCAAAATTAAAACGTGCAG	181
	GRX480-chip-r1	AGCTATGGGTGATGGTGCTG	
WRKY33	WRKY33-chip-f1	GGGTCAATTTGTAGCCTATCTCTA	713
	WRKY33-chip-r1	AAATCCATGTGCGGCTGTTT	
JAZ5	JAZ5-chip-f1	TACTGTTGCTAGTGCGTCGT	927
	JAZ5-chip-r1	TCTCCGATACGATCCGACAG	
JAZ5-3'	JAZ5-3'-f	TCC ATT TTA CGC GCA ATC CAC	
	JAZ5-3'-r	TCA GAT TTC TCC GGC GCT TG	
JAZ5-5'	JAZ5-5'-f	AAC CCG AGA CTA GCT ACA GCA	
	JAZ5-5'-r	AAT CAA TCT CCA TCT CCA ACT GTG	
JAZ7	JAZ7-chip-f1	GGCGAAAAATGTGTGGGTTCAACG	607
	JAZ7-chip-r1	GTAACGTCCTGCCAAACCCGTCC	
JAZ7	JAZ7-chip-f2	TGGCATTTCCCACGTGGATT	1550
	JAZ7-chip-r2	TGGTCGTCGCGTGATTTAGT	

JAZ8	JAZ8-chip-f1	TCATCAATCCCGTTTAACTCACA	957
	JAZ8-chip-r1	GTCTGACCATTCGCATTACGC	
JAZ8	JAZ8-chip-f2	TGGAACGTTACTTTCGGCTC	507, 625
	JAZ8-chip-r2	TGTGATCCATTGCTCACGCA	
MYC2	MYC2-chip-f2	GAATAACTCGAATTGGTCACATACA	1772
	MYC2-chip-r2	GGACATTAGTCGGGATTCGGG	
MYC2	MYC2-chip-f3	GTTTTCTAGTGGCGTCACCCCCAAAG	1475
	MYC2-chip-r3	CCAAACCATTGTCTGGTGGTTGTGAG	
