# Supplementary material for: LUX ARRHYTHMO mediates crosstalk between the circadian clock and defense in Arabidopsis
Source: Nat Commun. 2019 Jun 11;10:2543. doi: 10.1038/s41467-019-10485-6 (PMC6560066; doi:10.1038/s41467-019-10485-6)
Supplement: Supplementary file 3 — Reporting Summary [file 41467_2019_10485_MOESM3_ESM.pdf]

nature research

Corresponding author(s): Hua Lu

Last updated by author(s): Apr 29, 2019

Reporting Summary

Nature Research wishes to improve the reproducibility of the work that we publish. This form provides structure for consistency and transparency in reporting. For further information on Nature Research policies, see Authors & Referees and the Editorial Policy Checklist.

Statistics

For all statistical analyses, confirm that the following items are present in the figure legend, table legend, main text, or Methods section.

n/a	Confirmed	
☐	☒	The exact sample size (*n*) for each experimental group/condition, given as a discrete number and unit of measurement
☐	☒	A statement on whether measurements were taken from distinct samples or whether the same sample was measured repeatedly
☐	☒	The statistical test(s) used AND whether they are one- or two-sided<br>*Only common tests should be described solely by name; describe more complex techniques in the Methods section.*
☐	☒	A description of all covariates tested
☐	☒	A description of any assumptions or corrections, such as tests of normality and adjustment for multiple comparisons
☐	☒	A full description of the statistical parameters including central tendency (e.g. means) or other basic estimates (e.g. regression coefficient) AND variation (e.g. standard deviation) or associated estimates of uncertainty (e.g. confidence intervals)
☐	☒	For null hypothesis testing, the test statistic (e.g. *F*, *t*, *r*) with confidence intervals, effect sizes, degrees of freedom and *P* value noted<br>*Give P values as exact values whenever suitable.*
☒	☐	For Bayesian analysis, information on the choice of priors and Markov chain Monte Carlo settings
☐	☒	For hierarchical and complex designs, identification of the appropriate level for tests and full reporting of outcomes
☐	☒	Estimates of effect sizes (e.g. Cohen's *d*, Pearson's *r*), indicating how they were calculated

Our web collection on statistics for biologists contains articles on many of the points above.

Software and code

Policy information about availability of computer code

Data collection	The RNA-seq data were generated via the Illumina HiSeq sequencing platform. The clock activity data were collected by measuring the luminescence with an Omega Luminescence Reader.
Data analysis	The analyses of RNA-seq data and the circadian clock data were achieved by using available R packages.

For manuscripts utilizing custom algorithms or software that are central to the research but not yet described in published literature, software must be made available to editors/reviewers. We strongly encourage code deposition in a community repository (e.g. GitHub). See the Nature Research guidelines for submitting code & software for further information.

Data

Policy information about availability of data

All manuscripts must include a data availability statement. This statement should provide the following information, where applicable:

- Accession codes, unique identifiers, or web links for publicly available datasets
- A list of figures that have associated raw data
- A description of any restrictions on data availability

Source data are provided in the paper, its supplementary files, and a Source Data file for Figures 1B, 1D-1G, 2B-2G, 3B-3F, 4A-4D, 5B-5D, 5F-5H, 5J-L, Supplementary Figures 1, 2, 5, 6, 7, 8, and 9). The RNA-seq data are deposited in the National Center for Biotechnology Information Gene Expression Omnibus database with accession number GSE115680.

Field-specific reporting

Please select the one below that is the best fit for your research. If you are not sure, read the appropriate sections before making your selection.

☒ Life sciences ☐ Behavioural & social sciences ☐ Ecological, evolutionary & environmental sciences

For a reference copy of the document with all sections, see nature.com/documents/nr-reporting-summary-flat.pdf

Life sciences study design

All studies must disclose on these points even when the disclosure is negative.

Sample size	The sample size was chosen on the basis of prior studies that showed significant effects with similar sample sizes.
Data exclusions	Data from contaminated samples, e.g. contaminated seedlings and plates, were excluded.
Replication	Replicated experiments were successful and support conclusions drawn in this report.
Randomization	Samples were chosen randomly from each genotype per treatment per time point.
Blinding	Investigators were not blinded during experiments.

Reporting for specific materials, systems and methods

We require information from authors about some types of materials, experimental systems and methods used in many studies. Here, indicate whether each material, system or method listed is relevant to your study. If you are not sure if a list item applies to your research, read the appropriate section before selecting a response.

Materials & experimental systems

n/a	Involved in the study	
☐	☒	Antibodies
☒	☐	Eukaryotic cell lines
☒	☐	Palaeontology
☒	☐	Animals and other organisms
☒	☐	Human research participants
☒	☐	Clinical data

Methods

n/a	Involved in the study	
☒	☐	ChIP-seq
☒	☐	Flow cytometry
☒	☐	MRI-based neuroimaging

Antibodies

Antibodies used	Anti-GFP antibodies (Abcam, product code 290) and whole rabbit IgG (mock) (Jackson ImmunoResearch cat 011-000-003) coated onto Dynabeads Protein G (Invitrogen cat 100.04D) were purchased from indicated manufacturers.
Validation	The effectiveness of the antibodies was confirmed by the ChIP experiment that showed the binding of the LUX protein to several known LUX gene promoter targets, including the LBS sites in PRR7, PRR9, and GI promoters.
